# Patient-Reported Understanding of Emergency Department Discharge Instructions, Satisfaction, and Acceptability of a Future Telemedicine-Based Call-Back Program: Phase 1 Observational Pilot Study at a Tertiary Hospital in Dubai, United Arab Emirates

**DOI:** 10.7759/cureus.111949

**Published:** 2026-07-02

**Authors:** Maryam K Alrazooqi, Eman Almerashi Alhashmi, Firas AlNajjar

**Affiliations:** 1 Emergency Medicine, Rashid Hospital Trauma Center, Dubai, ARE

**Keywords:** call-back program, discharge instructions, ed revisits, emergency department, follow-up care, patient satisfaction, patient understanding, pilot study, telemedicine

## Abstract

Background

Emergency department (ED) revisits remain a significant global challenge, with ineffective discharge communication identified as a key contributing factor. Limited patient understanding of discharge instructions may negatively impact satisfaction, adherence to post-discharge instructions, and subsequent revisits to the ED. Telemedicine-based follow-up has emerged as a potential strategy to address this gap.

Objective

The aim of this study was to evaluate patient satisfaction and patient-reported understanding of ED discharge instructions and to assess the acceptability of a future telemedicine-based call-back program following ED discharge.

Methods

This prospective single-center observational pilot study was conducted at a tertiary ED in Dubai, United Arab Emirates. Adult patients discharged with non-life-threatening conditions were systematically selected and surveyed using a structured questionnaire assessing patient-reported understanding of discharge instructions across four domains (diagnosis, ED care, post-discharge care, and return precautions), satisfaction, and preference for a future telemedicine-based call-back program. Descriptive statistical analysis was performed using Microsoft Excel (Microsoft Corp., Redmond, WA, USA).

Results

A total of 100 patients were included. Overall patient-reported understanding and satisfaction were generally high across the cohort. Among the understanding domains, return precautions demonstrated the highest proportion of fair (n=19, 19%) and poor (n=8, 8%) responses. Overall satisfaction was generally high, with the largest proportion of participants reporting being moderately satisfied (n=34, 34%), followed by extremely satisfied (n=31, 31%) and quite satisfied (n=19, 19%). A total of 78 participants (78%) reported that a call-back by an emergency healthcare professional should be implemented in the future. Participants who reported that a call-back should be implemented demonstrated slightly lower understanding scores despite similar satisfaction levels.

Conclusion

This pilot study demonstrated generally high patient-reported understanding of and satisfaction with ED discharge instructions, as well as strong patient interest in a future telemedicine-based call-back program. These findings provide baseline data for the subsequent Phase 2 study, which will assess whether a structured telemedicine-based call-back program is associated with changes in patient-reported understanding, patient satisfaction, and ED revisit rates.

## Introduction

Frequent revisits to the emergency department (ED) remain an ongoing challenge in emergency medicine worldwide. Studies suggest that 18% of patients revisit the ED within 72 hours due to physician-related causes, with ineffective discharge instructions identified as the most common contributing factor at 56.7% [1]. This highlights the importance of effective discharge communication and patient education at the time of discharge. Ineffective discharge instructions may lead to poor patient satisfaction, non-adherence to follow-up recommendations or medications, and subsequent ED revisits [2]. 

Previous studies have shown that many patients do not fully understand ED discharge instructions. Engel et al. reported that only 13% of patients fully understood the four major components of discharge instructions: diagnosis and cause, care provided in the ED, care after the ED visit, and return precautions [3]. Sheikh et al. further demonstrated that poor comprehension of discharge instructions was more common among patients with lower socioeconomic status, suggesting the need for additional emphasis and reinforcement on discharge instructions, which can be done via follow-up phone calls [4].

Telemedicine-based follow-up has been proposed as a strategy to reinforce discharge instructions, clarify patient concerns, improve patient satisfaction, and potentially reduce ED revisits following discharge [2]. Fruhan et al. demonstrated significantly lower seven-day ED revisit rates among patients who received a telemedicine-based follow-up phone call within two days of discharge [5]. Patel and Vinson similarly demonstrated improved patient satisfaction among patients who received physician telephone follow-up after an ED visit [6]. 

This manuscript reports Phase 1, the observational pilot phase of a two-phase study evaluating a future telemedicine-based call-back intervention following ED discharge. The primary aim of this pilot was to assess patient-reported understanding of discharge instructions, evaluate patient-reported satisfaction with ED discharge instructions, and explore patient perspectives regarding implementation of a future telemedicine-based call-back intervention. Findings from this pilot will inform the design of a larger interventional study evaluating ED revisits, adherence to outpatient follow-up instructions and appointments, and post-discharge complications over a four-week follow-up period. 

Improving discharge communication may help optimize healthcare resource utilization, reduce unnecessary ED revisits, and identify patients requiring additional post-discharge support. To our knowledge, this represents one of the first resident-led telemedicine-based ED follow-up initiatives being developed and evaluated in the United Arab Emirates and the wider region.

## Materials and methods

Study design and setting

This manuscript reports the findings of Phase 1, a prospective single-center observational pilot study conducted in the ED of Rashid Hospital, a Level 1 trauma center in Dubai, United Arab Emirates. This pilot serves as the foundational observational phase to inform the subsequent Phase 2 interventional study evaluating a telemedicine-based call-back intervention.

Study population

Adult patients aged ≥18 years discharged from the ED with non-life-threatening conditions were eligible for inclusion. We defined non-life-threatening conditions as presentations where the patient remained hemodynamically stable, required no immediate life- or limb-saving interventions, and met all institutional clinical criteria for safe discharge home. Participants were required to provide informed consent. Patients with cognitive impairment or inability to provide consent, patients requiring hospital admission, and patients leaving against medical advice were excluded from the study.

Sampling and sample size

A total of 246 eligible patients were systematically screened and approached from daily ED discharge lists, whereby every fourth patient was evaluated according to predefined inclusion and exclusion criteria. Of these, 100 patients successfully completed the pilot study, and 146 declined or did not respond, resulting in a response rate of 40.7%. No formal sample size calculation was performed, as the sample size was selected pragmatically based on the pilot nature of the study. The pilot was exploratory in nature and intended to inform the design of a future interventional study rather than perform formal hypothesis testing.

Questionnaire administration and data collection

Electronic medical records were reviewed to confirm eligibility criteria. Eligible patients were electronically sent a structured self-administered questionnaire and informed consent forms (Appendices A-B) within 72 hours of ED discharge through Salama, the institution's Epic-based electronic medical record system, via the MyChart patient portal (Epic Systems Corporation, Verona, WI, USA). To reflect real-world clinical operations, questionnaire administration followed a pragmatic, asynchronous design. Electronic medical records were vetted through daily batch screening, and questionnaires were distributed at varying time points within the predefined 72-hour window depending on the timing of eligibility verification. Questionnaire completion times also varied according to when participants accessed the questionnaire through the patient portal. Nevertheless, all responses were obtained within the predefined 72-hour window to minimize recall bias while maintaining a pragmatic recruitment process. To optimize completion rates, a single electronic reminder notification was manually sent by the investigator through MyChart specifically to individuals who had already provided informed consent but had not yet completed the questionnaire. The questionnaire assessed patient-reported understanding across four domains of ED discharge instructions: diagnosis and cause, ED care provided, post-discharge care, and return precautions. The questionnaire also assessed overall satisfaction with discharge instructions using a Likert scale and explored patient perspectives regarding future implementation of a telemedicine-based call-back program. No active telephone call-backs or separate post-discharge interventions were performed during this pilot study phase.

The questionnaire was adapted from investigator-developed instruments used in previously published studies assessing patient understanding of emergency department discharge instructions, specifically the work of Engel et al. [3] and Sheikh et al. [4]. To evaluate the performance and internal reliability of the adapted instrument within our study population, psychometric analysis was performed. Moderate-to-strong positive correlations were observed between the questionnaire domains (r = 0.604-0.794), supporting the measurement of a common underlying construct. Additionally, the final four-item comprehension scale demonstrated excellent internal consistency reliability, with a Cronbach’s alpha of 0.904.

Data management

Data were anonymized and stored on encrypted systems with access restricted to study investigators. 

Statistical analysis

Data were analyzed using Microsoft Excel (Microsoft Corp., Redmond, WA, USA). Categorical variables were summarized as frequencies and percentages. Age was collected as predefined categorical age groups rather than exact continuous age values; therefore, numerical measures such as mean ± standard deviation (SD) or median (interquartile range (IQR)) could not be calculated. Age was summarized using frequencies, percentages, and the most frequently represented age group (mode). Likert scale responses were coded numerically from one to five, with higher scores indicating greater patient-reported understanding or satisfaction.

For understanding domains (Q1-Q4), Poor was coded as one, Fair as two, Good as three, Very Good as four, and Excellent as five. Satisfaction responses (Q5) were similarly coded from one (poorly satisfied) to five (extremely satisfied).

Demographic variables included age group, gender, and nationality. Outcome variables included patient-reported understanding of diagnosis (Q1), ED care (Q2), post-discharge care (Q3), return precautions (Q4), overall satisfaction with discharge instructions (Q5), and perspectives regarding implementation of a future call-back intervention (Q6).

Mean understanding and satisfaction scores were compared descriptively between participants who reported that a call-back by an emergency healthcare professional should be implemented in the future and those who did not. Exploratory relationships between understanding domains (Q1-Q4) were also assessed descriptively to evaluate consistency across patient-reported understanding domains.

No inferential statistical testing was performed due to the exploratory nature of the pilot study.

Ethical considerations

Ethical approval was obtained from the Mohammed Bin Rashid University of Medicine and Health Sciences Institutional Review Board (Approval No. MBRU IRB-2024-287) prior to study initiation. Written informed consent was obtained from all participants prior to enrollment. Confidentiality and data protection were maintained throughout the study.

## Results

Participant demographics

The pilot cohort was predominantly male (n=62, 62%), with females comprising 38 participants (38%). The participant age distribution was as follows: 18-25 years (n=23, 23%), 26-30 years (n=14, 14%), 31-40 years (n=33, 33%), 41-59 years (n=21, 21%), and ≥60 years (n=9, 9%). The most frequently represented age group (mode) was 31-40 years (n=33, 33%). Participants represented a wide range of nationalities, with the largest proportion from the United Arab Emirates (n=28, 28%), followed by India (n=15, 15%) and Pakistan (n=11, 11%), reflecting the diverse population served by the ED.

Overall understanding of ED discharge communication

Across the four understanding domains (Q1-Q4), most participants reported good, very good, or excellent understanding. ED care (Q2) had the highest proportion of excellent responses (n=36, 36%), followed by diagnosis (Q1; n=33, 33%). Post-discharge care (Q3) had the highest proportion of good responses (n=42, 42%). Return precautions (Q4) demonstrated the highest proportion of fair responses (n=19, 19%) and poor responses (n=8, 8%).

The distribution of patient-reported understanding across the four domains is shown in Table 1.

**Table 1:** Distribution of patient-reported understanding across four domains of emergency department discharge communication.

Understanding Domain	Excellent n (%)	Very Good n (%)	Good n (%)	Fair n (%)	Poor n (%)
Diagnosis (Q1)	33 (33%)	23 (23%)	29 (29%)	11 (11%)	4 (4%)
ED care (Q2)	36 (36%)	22 (22%)	26 (26%)	11 (11%)	5 (5%)
Post-discharge care (Q3)	28 (28%)	14 (14%)	42 (42%)	12 (12%)	4 (4%)
Return precautions (Q4)	26 (26%)	20 (20%)	27 (27%)	19 (19%)	8 (8%)

Satisfaction with discharge instructions

Overall satisfaction with discharge instructions (Q5) was generally high. The largest proportion of participants reported being moderately satisfied (n=34, 34%). This was followed by 31 participants (31%) who were extremely satisfied and 19 (19%) who were quite satisfied. Lower satisfaction levels were reported by nine participants (9%) who were a little satisfied and seven participants (7%) who were poorly satisfied.

Patient satisfaction levels following emergency department discharge instructions are shown in Table 2.

**Table 2:** Distribution of patient satisfaction levels following emergency department discharge instructions.

Satisfaction Level	Count (n)	Percentage (%)
Extremely satisfied	31	31%
Quite satisfied	19	19%
Moderately satisfied	34	34%
A little satisfied	9	9%
Poorly satisfied	7	7%
Total	100	100%

Perspectives regarding future implementation of a call-back intervention 

A total of 78 participants (78%) reported that a call-back by an emergency healthcare professional should be implemented in the future, while 22 participants (22%) did not. 

Comparison by future call-back implementation preference

Mean understanding and satisfaction scores for participants who reported that a call-back should be implemented in the future and those who did not are presented in Table 3.

**Table 3:** Comparison of mean understanding and satisfaction scores according to perspectives regarding future implementation of a call-back intervention.

Question	Yes to Call-Back (Mean)	No to Call-Back (Mean)
Q1. Understanding of the diagnosis	3.65	3.86
Q2. Understanding of ED care	3.72	3.77
Q3. Understanding of discharge instructions	3.46	3.64
Q4. Understanding of return precautions	3.38	3.32
Q5. Overall satisfaction	3.58	3.64

Participants who supported future implementation demonstrated slightly lower mean understanding scores for diagnosis (Q1: 3.65 vs 3.86) and post-discharge instructions (Q3: 3.46 vs 3.64). Mean understanding scores for ED care (Q2) were similar between groups (3.72 vs 3.77), and understanding of return precautions (Q4) was comparable (3.38 vs 3.32). Mean satisfaction scores (Q5) were also similar between groups (3.58 vs 3.64).

## Discussion

Patient understanding of discharge instructions

The overall patient-reported understanding observed in this study appeared higher than that reported in much of the international literature. Only four to eight participants (4%-8%) reported poor understanding across the four assessed domains, whereas substantially greater deficits in understanding have been described elsewhere. Engel et al. reported substantial knowledge deficits following ED discharge, with 80% of patients demonstrating deficits in home care instructions and 79% in return instructions. More than half of patients demonstrated minimal or no understanding of return precautions [7]. Similarly, a systematic review and meta-analysis by Hoek et al. reported that only 47% of patients correctly recalled verbal discharge instructions [8]. Comparable findings have been reported by Kessels, who demonstrated that patients forget between 40%-80% of medical information immediately after consultation, with nearly half of retained information being inaccurate [9].

These findings highlight an important gap between discharge communication delivered by healthcare professionals and information retained or understood by patients after leaving the ED. Several factors may contribute to this gap. Variations in discharge practices, written instruction quality, patient health literacy, and healthcare system organization may influence patient-reported understanding.

Despite the generally favorable results, return precautions demonstrated the greatest variability among the understanding domains, with 19 participants (19%) reporting fair understanding and eight participants (8%) reporting poor understanding. This finding is consistent with patterns described in previous literature. A recent meta-analysis involving adults discharged from EDs demonstrated significant discrepancies in understanding of return precautions, suggesting this remains a particularly challenging component of discharge communication [10]. Supporting this, observational data from two large academic EDs in the United States demonstrated that return precautions were included in only 54% of verbal discharge communications and that understanding was assessed in only 53% of discharges overall [11]. Similarly, Samuels-Kalow et al. highlighted persistent gaps in patient understanding of return instructions, even when other discharge domains are well understood. This is clinically significant, as inadequate understanding of return precautions has been directly associated with delayed re-presentation and adverse outcomes [12]. These findings support the need for discharge strategies that reinforce not only diagnosis and treatment, but also when and why patients should return to the ED. 

The relatively high patient-reported understanding observed in this study may also reflect contextual and population-related factors. The ED serves a diverse multinational population, with the largest participant groups originating from the United Arab Emirates, India, and Pakistan. Differences in baseline health literacy, family involvement in care, and expectations of clinician communication may differ from those described in predominantly Western populations. Engel et al. demonstrated marked discrepancies between patients’ perceived and actual understanding of discharge instructions, emphasizing the importance of objective assessment tools when evaluating discharge communication quality [7]. Similarly, Griffey et al. found that teach-back did not improve patient satisfaction or perceived comprehension, but did improve comprehension of post-discharge care, medications, self-care, and follow-up instructions among patients with limited health literacy, further emphasizing that perceived understanding may not accurately reflect actual comprehension [13]. Prior research has demonstrated that patients may not recognize gaps in their own understanding of discharge instructions, highlighting the limitation of relying solely on self-assessment measures [12].

Perspectives on future call-back implementation

The high level of patient interest in future implementation of a call-back intervention observed in the present study, with 78 participants (78%), suggests substantial interest in structured post-discharge follow-up after ED discharge. Participants who reported that a call-back by an emergency healthcare professional should be implemented in the future demonstrated slightly lower understanding scores despite similar satisfaction levels. Taken together, these findings suggest that interest in a future call-back intervention may be due to residual uncertainty, a need for clarification, or a desire for reassurance rather than dissatisfaction alone. This finding is consistent with previous literature suggesting that some patient groups may require additional reinforcement of discharge instructions following ED discharge [4].

The rationale for post-discharge follow-up is closely linked to the challenges of information retention and understanding identified above. If patients may forget, misunderstand, or remain uncertain about key discharge information after leaving the ED, a structured call-back provides an opportunity to reinforce instructions, clarify concerns, and identify patients who may require additional guidance.

Several previous studies have demonstrated the potential benefits of structured post-discharge telephone follow-up interventions. Fruhan and Bills demonstrated significantly lower seven-day ED revisit rates among patients receiving callbacks at two days post-discharge compared with controls [5]. Similarly, Harrison et al. demonstrated reduced 30-day readmission rates following structured post-discharge telephone follow-up interventions [14]. Patel and Vinson also demonstrated improved patient satisfaction among patients who received physician telephone follow-up after an ED visit [6].

Nevertheless, evidence regarding universal callback interventions remains mixed. Boggan et al. found no reduction in 30-day ED revisits or hospital readmissions following post-discharge contact in a systematic review and meta-analysis of 13 studies [15], and Yiadom et al. similarly demonstrated no significant impact on 30-day readmissions [16]. Mistiaen and Poot found that telephone follow-up interventions improved patient satisfaction and perceptions of continuity of care, although effects on readmissions and healthcare utilization were less consistent [17]. Taken together, the literature suggests that call-back interventions may be most useful when they are structured, targeted, and focused on reinforcement of discharge instructions rather than implemented as a non-specific follow-up process.

The findings of the present study support further evaluation of a structured telemedicine-based call-back intervention in ED populations. In particular, the observed variability in return precaution understanding and the high level of patient support for future implementation suggest that post-discharge call-backs may provide an opportunity to reinforce discharge instructions and address residual patient uncertainty following ED discharge. To our knowledge, this represents one of the first resident-led telemedicine-based ED follow-up initiatives being developed and evaluated in the United Arab Emirates.

Limitations

This study has a few distinct limitations. First, all findings relied on patient-reported understanding and satisfaction rather than objectively verified comprehension. Because these metrics are self-reported, they reflect subjective patient understanding, which may overestimate true comprehension. Additionally, the questionnaire was administered in English only. In a highly multinational patient population such as ours, this may have influenced participant comprehension and response accuracy. Furthermore, preferred language and baseline English proficiency were not specifically assessed. Although eligible patients were identified using systematic sampling, questionnaire participation was voluntary; therefore, non-response bias cannot be excluded, as patients who chose to complete the questionnaire may have differed systematically from those who did not respond. Finally, as a single-center study conducted in a tertiary ED in Dubai, the findings may not be generalizable to other healthcare settings or populations. 

## Conclusions

ED discharge communication in this Phase 1 observational pilot study was generally well received by patients, with high levels of patient-reported understanding and satisfaction. However, understanding of return precautions demonstrated greater variability compared with other discharge domains. A substantial proportion of participants reported that a call-back by an emergency healthcare professional should be implemented in the future, indicating acceptability of a future telemedicine-based call-back intervention following ED discharge. These findings provide baseline data for the subsequent Phase 2 study, which will assess whether a structured telemedicine-based call-back program is associated with changes in patient-reported understanding, patient satisfaction, and ED revisit rates.

## Figures and Tables

**Figure 1:**
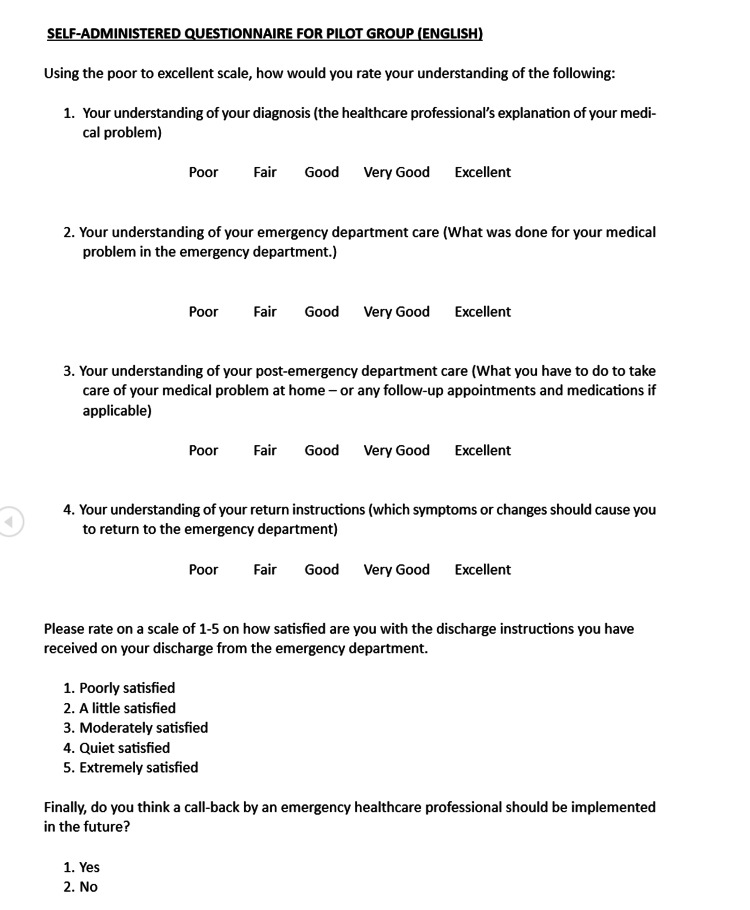
Pilot study questionnaire The following questionnaire was electronically administered to eligible participants within 72 hours of ED discharge as part of the observational pilot phase of the study.

**Figure 2:**
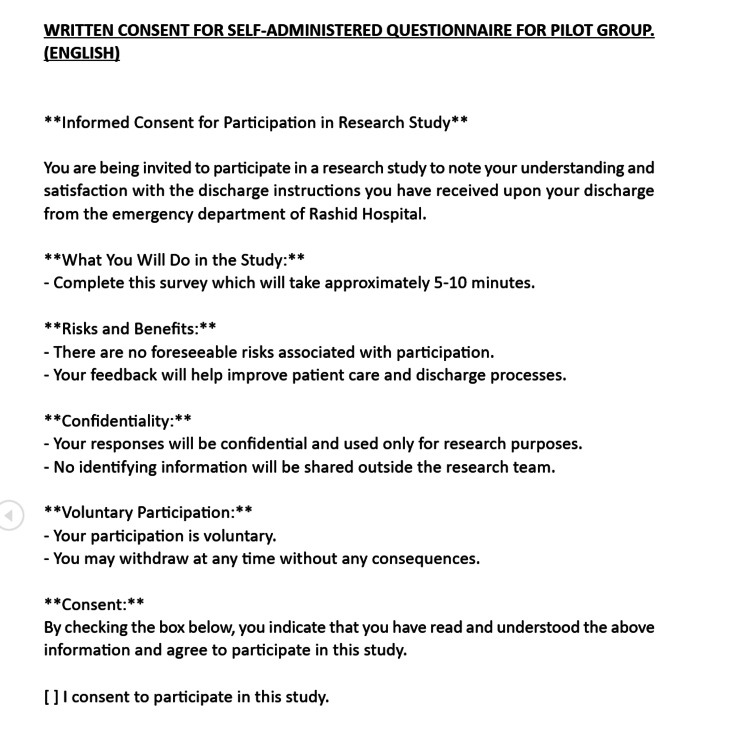
Electronic informed consent form The electronic informed consent form provided participants with information regarding the study purpose, voluntary participation, confidentiality, questionnaire administration process, and contact information for the study investigators. Electronic informed consent was obtained from all participants prior to questionnaire completion.

## Appendices

Appendix A 

Appendix B 
